# Supportive and non-supportive interactions in families with a type 2 diabetes patient: an integrative review

**DOI:** 10.1186/s13098-017-0256-7

**Published:** 2017-07-21

**Authors:** Birgitte B. Bennich, Michael E. Røder, Dorthe Overgaard, Ingrid Egerod, Lene Munch, Filip K. Knop, Tina Vilsbøll, Hanne Konradsen

**Affiliations:** 1 Center for Diabetes Research, Gentofte Hospital, University of Copenhagen, Hellerup, Denmark; 2 Institute of Nursing, Metropolitan University College, Copenhagen N, Denmark; 3 Intensive Care Unit, Copenhagen University Hospital Rigshospitalet, Copenhagen, Denmark; 4 Faculty of Health & Medical Sciences, University of Copenhagen, Copenhagen, Denmark; 5 Department of Clinical Medicine, Faculty of Health and Medical Sciences, University of Copenhagen, Copenhagen, Denmark; 6 NNF Center for Basic Metabolic Research, Faculty of Health and Medical Sciences, University of Copenhagen, Copenhagen, Denmark; 7 Steno Diabetes Center Copenhagen, University of Copenhagen, Copenhagen, Denmark; 8 Department of Neurobiology, Care Sciences and Society, NVS, Karolinska Instituttet, Alfred Nobels Allé 23, 141 52 Hundinge, Sweden

**Keywords:** Family, Interaction, Literature review, Support, Type 2 diabetes, Involvement

## Abstract

**Background:**

Type 2 diabetes and its management affect the patient and the close family potentially causing either psychological distress or increased sense of responsibility and collaboration in these families. Interactions between patient and family play an important role in maintaining lifestyle changes and diabetes self-management. The purpose of this integrative review was to summarise and assess published studies on the intra-family perspective of supportive and non-supportive interactions in families with a type 2 diabetes patient.

**Methods:**

Included in the review were published qualitative and quantitative studies that examined the intra-family perspective on supportive and non-supportive interactions. We searched the literature from 2000 to 2016 and the search strategy comprised the following databases: Cochrane, PubMed, CINAHL, Web of Science, PsycINFO and Psyc-ARTICLES as well as hand searching of reference lists. Quality assessment, data extraction and analysis were undertaken on all included studies.

**Results:**

We identified five eligible research papers. Employing content analysis three categories describing interactions were refined: Impact of practical action, impact of emotional involvement, and impact of communication content. Supportive interactions included encouraging communication and family collaboration in managing diet, medications, and blood glucose checking. Non-supportive interactions were visible irritation, nagging behaviour and refusing to share the burden of living with diabetes.

**Conclusion:**

The findings stress the importance of including both patient and family in clinical practice to target diabetes self-management adherence and well-being of the whole family. The majority of self-management occurs within the family environment. Therefore, the intra-family perspective of supportive and non-supportive interactions should be understood and addressed as the family members are interdependent and affected by each other. Future research assessing the impact of professional support and the family function will have the potential to improve the daily life and well-being of patients with type 2 diabetes as well as the whole family.

## Background

Diabetes affects around 415 million people worldwide, most of whom are diagnosed with type 2 diabetes. In 2011–2012, the estimated prevalence of diabetes was 12–14% in US adults and the prevalence is increasing in most countries [1, 2]. The aetiology of type 2 diabetes involves genetic as well as environmental components, including socioeconomic risk factors [3]. Type 2 diabetes is a progressive disease associated with risk of microvascular complications (i.e. retinopathy, nephropathy and neuropathy), macrovascular disease (i.e. stroke, myocardial infarction and peripheral artery disease) and premature death. Management of type 2 diabetes includes lifestyle changes and intensification of medication over time to maintain glycaemic control and thus reduce the risk of microvascular and macrovascular complications [4]. Additionally, most patients with type 2 diabetes are overweight or obese with hypertension and dyslipidaemia, often requiring multi-pharmacological treatment. Despite advances in diagnostics and treatment, many patients still experience inadequate glycaemic control related to poor adherence to behavioural and pharmacological recommendations. Important reasons for non-adherence are self-management challenges (e.g. healthy diet, exercise, blood glucose checking, pharmacological treatment), clinicians’ inadequate intervention strategies, conflicting views on life versus disease and disagreement regarding the patient’s health status [5–8].

Many patients with type 2 diabetes experience psychological issues affecting their ability to cope and manage their disease. Unfortunately, healthcare providers, including nurses, often report lack of resources to provide sufficient support [9, 10]. During short and busy consultations nurses and physicians often focus on aetiology, diagnosis, pathophysiology and treatment of the disease, while patients are more concerned with the consequences and impact on daily life and family relations [5–7, 11, 12]. Multiple approaches to family interventions as to improve diabetes self-management have been examined [13]. However, theoretical knowledge about family theory and family-based education seem to be lacking among diabetes educators influencing the impact of the intervention [14].

Moreover, the patients’ perceptions of support or lack of support usually refer to the family [15–17]. Interactions between adult patients and their family play a major role in maintaining lifestyle changes and optimising diabetes self-management. Thus, family support regarding meal-planning, medication reminders, glucose checking and exercise affects the patient’s self-management adherence and the well-being of both the patient and their family [15–19]. In addition, good family function is associated with adequate patient support [20]. Family members are interdependent as they react to each other’s needs and concerns, thus, acknowledging individual reactions promotes a sense of responsibility and family cohesion [17, 21, 22].

Type 2 diabetes affects family members differently, either by improving family cohesion or causing psychological distress. In some families the obligation to support the patient is experienced as a burden [23]. Close family members, particularly spouses, are affected by changes in the patient’s health and need to know how to provide the best support [24]. Moreover, disruptive family behaviours, such as bickering about diet, exercise or medications are barriers to the patient’s effective self-management [25, 26]. It is worth noting that non-supportive interactions have a relatively stronger impact on self-management than supportive interactions [20, 23].

More evidence on how health professionals might effectively tap the potential of supportive family interaction and prevent non-supportive behaviour is needed [13, 27, 28]. A relationship between social support and diabetes self-management adherence is found, but being able to examine the potential of family interventions, focusing on the family dynamics, requires a more detailed exploration of supportive and non-supportive interactions in the perspective of the family [29].

The aim of this integrative review was to identify, assess and summarise published studies providing an intra-family perspective to supportive and non-supportive interactions in families with a type 2 diabetes patient.

## Methods

Our review had a multiple methods design as described by Whittemore and Knafl [30]. An integrative review is considered the broadest type of review and allows for the inclusion and combination of diverse methodologies and presentation of a variety of perspectives on the phenomenon of interest [31, 32]. Strategies include specifying the purpose, searching the literature, analysing and synthesising data, and finally, evaluating and presenting results [30].

We searched the following databases: Cochrane, PubMed, CINAHL, Web of Science, PsycINFO and Psyc-ARTICLES as well as hand searching of reference lists, and structured the search by Patient, Interest and Context, PICo [33]. We used the following keywords for Patient (P): ‘Diabetes mellitus type 2 or ‘NIDDM’, for the phenomena of Interest (I) ‘interaction’ or ‘function’ or ‘connection’ or ‘behaviour’ or ‘support’ or ‘relation’ or ‘psychosocial’ or ‘illness perception’ and for Context (Co) ‘family’ or ‘caregiver’ or ‘significant other’ or ‘relatives’ or ‘carer’ or ‘spouse’ or ‘couples’ or ‘partner’. The limits were set to English language, publications in 2000–2016, as to cover recent research, and adults 18 years or older.

Inclusion criteria were: (1) families with a member with type 2 diabetes, (2) focus on supportive and/or non-supportive intra-family interactions related to life with type 2 diabetes. Exclusion criteria were: (1) non-western culture, (2) studies focusing only on either the patient or family perspective.

We have adhered to the following family construct: “Family members are not necessarily marital or blood-related, but could be neighbours or good friends” [34]. Social interaction in the family was broadly defined as a symbolic, mutual exchange between two or more individuals with a common or shared history, in which information is communicated both verbally and nonverbally [35]. The intra-family perspective with particular focus on interaction and reciprocity was chosen to concentrate on the family as the unit of care, which has been termed the “we-ness” [36].

The first author (BBB) conducted the searches from 2000 to 2016 in collaboration with an information specialist to increase reliability. Papers found were excluded on the basis of titles or abstracts where the focus was exclusively on either the patient or family, provided a non-western cultural perspective or were dissertations. Full-text articles were assessed for eligibility and further excluded when lack of intra-family perspective, yielding five articles for our review. These five articles were quality assessed using the Critical Appraisal Skills Programme, CASP [37]. All studies included used a variety of descriptive methodologies and non-comparable quality assessment criteria. Therefor we decided not to exclude studies by quality.

Data were structured in a matrix. The first (BBB) and last author (HK) performed the data abstraction. Findings extracted from the articles were synthesised using content analysis, as the objective was to describe and understand data [38, 39]. Content analysis was chosen because of its applicability in similar studies [40]. After reading the papers, the following question guided analysis: what characterises supportive and non-supportive interactions? Relevant data were extracted from each primary source after which meaning units were identified, condensed and labelled with a code referring to the context and maintaining the core information. An example is given in Table 1. The categories describing interactions included: impact of practical action, impact of emotional involvement, and impact of communication content (Table 3).

**Table 1:** Example of abstracting the content in the text into categories

Meaning unit	Condensed meaning unit	Code	Category
“When we go out to dinner or we go on trips or anything like that, he is even stricter than me sometimes. He’ll say, ‘well my wife can’t do that’. That helps me cope with the situation, and he is very happy to stay here at home where I can fix meals that I can eat” [41]	Help coping with the situation, stay at home to prepare meals	Preparing meals at home	Impact of practical actions

Codes were compared for similarities and differences and abstracted into the construction of categories. The categories are the manifest expression of the context, ‘what the text says’ [39]. This process of analysis moves back and forth from the whole text to its parts. Tentative categories were discussed to capture supportive and non-supportive family interaction among the authors using author triangulation to identify the final categories.

## Results

**Fig. 1:**
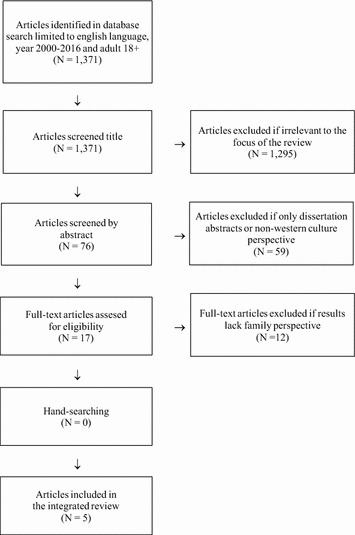
Flow diagram of study selection and inclusion

The search identified 1371 papers. 1366 were excluded related to criteria, see Fig. 1.

Included in the review were five articles representing results from three qualitative and two quantitative studies (Table 2).

**Table 2:** Summary of the included studies

Author and title	Country of origin	Aim of study, design and data	Sample size and characteristics	Main results
Trief et al. [41]. Describing support: a qualitative study of couples living with diabetes	USA	To learn about support from couples who deal with diabetes daily Grounded Theory and telephone interviews	40 patients (55% type 2 diabetes) and 32 spouses. Mean age patient and spouse 49 years	Three broad topics on spousal behaviour and diabetes management: (1) being helpful, especially regarding diet, general support and reminders, (2) being non-helpful, including nagging, wrong diet, poor communication and conflicts, (3) couple interaction as teamwork, independence, emotional support
Sandberg et al. [42]. “He said, She said”: the impact of gender on spousal support in diabetes management	USA	To examine how gender is related to support for couples coping with diabetes Grounded Theory and individual interviews	40 patients (55% type 2 diabetes) and 32 spouses. Mean age patient and spouse 49 years	Both males and females recognised the importance of spousal support. Males used more authoritative language, females more accommodating and collaborative. Females preferred verbal support, males instrumental. Females identified own nagging, males silent
Stephens et al. [45]. Spouses’ attempt to regulate day-to-day dietary adherence among patients with type 2 diabetes	USA	To investigate daily dietary adherence in older adults with type 2 diabetes as a function of spouses’ diet related support and control Quantitative study using diaries and questionnaires	126 couples where one partner had type 2 diabetes. Mean age 66 years	Spousal dietary support increased patient adherence, whereas persuasion and pressure decreased adherence. Support decreased distress and pressure increased distress and decreased adherence
Houston-Barrett et al. [43]. Couple’s relationship with diabetes: means and meanings for management success	USA	To develop a theory about dyadic constructs and their interactions with success in diabetes management Grounded Theory and dyadic interviews	25 couples, one with type 2 diabetes, mean age men 57 and women 55 years	Relationships with diabetes: (1) transforming, (2) accepting, (3) rejecting. Couple’s relationship to diabetes determines success. Focus on emotion and meaning leads to growth, accept or rejection
Dimitraki et al. [44]. The association of type 2 diabetes patient and spouse illness representation with their well-being: a dyadic approach	Greece	To examine type 2 diabetes in patients and spouses in relation to physical and psychological well-being Quantitative study using questionnaires	84 couples where one partner had type 2 diabetes. Mean age patients 65 and spouse 63 years	Synergy between patient and spouse; patients had anxiety and depression when spouse perceived diabetes as unpredictable. Spouses had anxiety when patient perceived diabetes less burdensome

Data collection methods in the studies included individual and dyadic interviews, diaries and questionnaires. In all of the studies, a spouse or partner represented the family. We will use the term partner in the following to lighten the prose. The mean age of the informants was 49–64 years. Supportive interactions in the families were defined in the studies according to a common goal of maintaining the patients’ adherence to the lifestyle recommended for diabetes or maintaining the physical and psychological well-being of all participants (patient and partner) (Table 3).

**Table 3:** Categories of family interactions when living with diabetes

Supportive	Non-supportive
*Impact of practical actions*	
Family assistance with grocery shopping, cooking, sharing and learning about diet plan [41–43] Adherence to dietary guidelines, timing the meals according to insulin, preparing meals at home, assisting with medications, assisting with checking blood sugar level [41, 42]	Spouse and patient buy or prepare non-healthy food, feel obliged to eat unhealthy food prepared by either the patient or spouse [41, 42]
*Impact of emotional involvement*	
Thinking of the others’ needs and concerns [41] Being cooperative [41] Making the other feel like a person [41] Positive attitude toward diabetes [41, 43] Acknowledge need for independence [41] Being calm [41] Acknowledge need for privacy [42] Take responsibility [44] Acknowledge responsibility for shared management [45] Have a shared construction of meaning [44] Be partners and work together [44]	Partner refusing to share burden with spouse, expecting patient to manage tasks alone rather than together, refusing to accept requirements and consequences of diabetes, focusing solely on problems [41, 43] Being scared and nervous [41] Prefer to remain uninvolved [44] Perceive diabetes as unpredictable and burdensome [45]
*Impact of communication content*	
Acting as a sounding board, talking nicely, reminding to check blood glucose, take medication, bring snacks, taking time to listen [41] Console, encourage, be there, reminding [42] Partners recognise the need of help in crisis situation [41] Asking how the other feels [43]	Nagging, criticising, constant controlling reminders, poor communication [41, 42] Being silent, ignoring the other, not communicating about difficulties, conflicts not relating to diabetes [41, 42] Most talk is about difficulties [44] Spouses get annoyed, aggravated and angry, difficult receiving help when hypoglycaemic-get agitated [41] Use persuasion or challenge food choices [43] Express irritation or doubt food choices [43] Telling each other what to feel [41, 42] Both preoccupied with reminders vs. nagging [41, 42]

### Impact of practical actions

Supportive actions were seen when patient and partner collaborated in practical activities such as maintaining a diet, shopping, cooking and mealtimes; including learning about dietary recommendations [41], dietary restrictions [42], and the importance of adhering to the recommendations [41, 43]. Supportive actions were also the coordination of mealtimes with work, exercise, medication administration [41], and even with social activities, when eating out [42].

Non-supportive actions were seen in cases of destructive behaviour, such as the deliberate preparation of non-healthy food [41, 42], or disregarding the coordination of mealtimes according to the diabetes schedule [42].

### Impact of emotional involvement

There is a delicate balance between the experience of involvement as supportive or non-supportive. The need for privacy and independence [41] was as strong as the need for shared responsibility when collaborating on diet, medication administration and blood glucose checking. It was important for the couples to have a shared understanding of life and goals with diabetes to avoid conflicts and misunderstandings [44].

The patient’s well-being was related to the partner’s emotional involvement and improved if the partner perceived diabetes as less burdensome and less unpredictable [45]. Persuasion and reminders were considered supportive as they were signs of emotional involvement [44]. Emotional involvement in each other’s feelings, needs and concerns, being cooperative and having a positive attitude toward diabetes was considered supportive [41, 43].

The non-supportive behaviour included lack of emotional involvement, pressure [44], and refusal to share the burden of the disease. Lack of emotional involvement was seen when the partner interfered with patient autonomy, refused to live with restrictions and focused exclusively on the negative aspects of the disease [41, 43].

Patients were affected by their own and their partners’ understanding of illness, whereas partners were mostly affected by their own situation unless the patient’s illness was perceived as serious [44]. Both patient and partner were challenged by situations of hypoglycaemia, which affected their relationship. Some partners were annoyed, aggravated or angry when the patient’s behaviour interfered with their daily routine or caused them embarrassment. If the patient became angry or prevented the partner in participating in the adjustment of blood glucose, the partner resorted to non-supportive communication, e.g. by forcing the patient to eat. Patients were emotionally upset in situations of low blood glucose, which was made worse by the partner’s anger and pressure [41]. Lack of emotional involvement could have a long-term impact the relationship between patient and partner [42].

### Impact of communication content

Emotional support was fundamental to couples, including open communication about feelings and ability to solve problems as a team [42, 43]. Supportive involvement included communication about keeping healthy foods at hand, checking blood glucose, taking medications, timing meals [42], eating snacks, carrying a cell-phone, sharing goals for diabetes management and gentle conversation within the family [41]. Supportive communication was experienced when the partner acted as a sounding board when difficult issues were discussed [41].

Non-supportive communication was experienced as nagging and increased frequency of reminders. Frequent reminders were experienced as control and/or critique [41, 43]. Nagging, verbal expressions of irritation, distrust in the dietary recommendations, or coercing the patient to stray from recommended diet [43] was described as non-supportive communication [41, 42]. Silence, lack of acknowledgement and conflicts not relating to diabetes also impeded diabetes management. Persistent nagging followed by silence prevented sound communication [41].

The male patients and partners regarded help with blood glucose management as supportive, whereas female patients and partners described communicative behaviour as supportive, e.g. asking questions, explaining behaviour and giving advice. Both female and male partners, however, provided verbal and instrumental support [41–43].

In our sample, supportive communication varied according to gender. Male partners predominantly used commands such as: ‘I watch her and tell her what she should eat and what not to eat’ [42], whereas female partners often used more accommodating language such as ‘I just try to ask him if he is balancing his meals right’ [42].

## Discussion

In this review, we present categories describing characteristics of supportive and non-supportive interactions in families where one member is diagnosed with type 2 diabetes. The main categories were interrelated as seen when the supportive and non-supportive interaction was described by different kinds of social control, regulation, influence and monitoring of an individual’s health behaviour.

The main finding in this review is the importance of the family in the management of type 2 diabetes. We found that collaborating, as a couple with shared goals, was considered supportive. It has been shown that a lack of support of patients’ self-care behaviour may impede patients’ efforts to implement the necessary behavioural changes [46]. Therefore, when family members sustain the patient’s self-management, they can be considered as facilitators and supportive [47]. Moreover, family support has a greater impact on self-reported diabetes coping than support from professionals [15].

Family as a source of support has mainly been described in relation to couples and gender differences. The partner represents the most frequently reported source of social control and support for married patients. Conversely, single men are supported by neighbours and friends, and single women are supported by their children [48]. Furthermore, married men reported the highest level of social control (persuasion and pressure) from their wives, followed by married women, single women and single men. Married men benefited most from their partners’ support on their diabetes management [48]. Children played a supportive role, especially for divorced or widowed mothers, whereas unmarried men often failed to receive sufficient social support to improve their health behaviour [49, 50]. Thus, emphasising the importance of family and partners in diabetes management.

Consistent with other studies [48, 51] we found that persuasion, as a strategy for social control, was supportive and efficient in promoting dietary behaviour change among married patients [42, 44]. This might be related to the difficulty of hiding dietary health behaviours when sharing most meals [41]. Patients tend to avoid discussing their disease due to experiences of prejudice and negative reactions, in particular in relation to comments about eating habits and diabetes being self-inflicted as a result of lifestyle choices [17]. Family and friends sharing meals with patients who are single are not apt to attempt social control or interfere with dietary choices [41].

Like prior studies, we found that social control leads to gratitude or hostility depending on how support is given and received by partner and patient. Patients of either gender appreciated frequent social support, but it was highly pronounced in women [51, 52]. Being able to communicate, help, and share responsibility was found to be supportive in our study. Earlier studies have shown that from a patient perspective, spousal support leads to less stress, better marital interaction and stronger adherence to diabetes management [48, 53]. By contrast, spousal coercion or pressure often leads to patient resentment regarding diabetes management as it is humiliating and undermines the patient’s sense of autonomy [48, 51, 52, 54].

Pressure, criticism, nagging and other negative behaviours were experienced as non-supportive interactions in our findings. This result is supported by other studies showing that forceful behaviour leads to a negative emotional response without a positive effect on health behaviour [41, 48, 51, 55]. Pressure, as social control or support, has also been shown to lead to distress, anxiety and low self-esteem, and is perceived by the recipient as targeting control rather than the well-being of the patient [52]. Family members should only take control in acute situations, where the patient is threatened by low blood glucose levels [16], which is consistent with our findings.

### Clinical implications

The importance of including the entire family in caring for patients with type 2 diabetes, raise implications for healthcare professionals in all sectors. The family members’ knowledge worries and attitudes should be understood and addressed since the majority of self-management occurs within the family environment. There is growing evidence towards both patients’ and family members’ improved clinical and psychosocial outcomes, when a family-oriented approach is undertaken by healthcare professionals in relation to chronic diseases, including diabetes [13, 56–58].

One approach, which is feasible in time restricted health care settings [59] is to involve families by brief family interviews as used in Family Systems Nursing [60]. Families’ responses after participation in Family Systems Nursing interventions, have been related to improvement of understanding and capability; caring more about each other and the family; family emotional well-being; individual emotional well-being; interactions within and outside the family and healthier individual behaviour. These findings meet the potentials and challenges of support outlined in our results [61].

However, interventions toward families of the chronically ill patients varied in scope, design, content and level of family involvement and with no evidence of long-term effects. Consequently, a firm determination of family interventions to improve outcomes for the patient and their family is lacking [13]. Therefore, more studies with good quality experimental designs and ample sample size are needed to strengthen the evidence base. Furthermore, the interventions need to be tailored to the culture, family structure and health beliefs of the patient [62]. Interventions could include supportive communication techniques and recognise the interdependence of family members as interaction affects them both [13, 20]. A clear description of the role of family, the extent of participation in the intervention and a specific target for self-management improvement is required to test long-term implications of clinical and psychosocial outcomes [13, 63].

### Strengths and limitations

Our review included literature using both qualitative and quantitative data. The use of multiple methods increases the validity when different types of data converge toward similar results, while at the same time results are limited by the small numbers of studies included and the descriptive nature of all included studies. The review was strengthened by multiple abstraction checks and the collaboration among authors. As with other reviews, our findings inherently rely on the quality of the studies included. We included studies that were conducted in similar contexts to strengthen the evidence and comparability. The findings of Trief et al. [41] and Sandberg et al. [42] originated from the same data source, viz. a population of 55% type 2 diabetic patients, which might affect our findings. We did not search for unpublished studies or studies published in books or non-indexed journals although this might have enriched findings in this review.

The knowledge that family support is essential in diabetes management does not necessarily imply that stronger family relations improve adherence in families or in general. The family dynamics described in this review are probably not limited to families with diabetes, except the situations caused by hypoglycaemia. Thus, our results are potentially relevant to families with other chronic diseases where adherence to a particular lifestyle is recommended. This is a potentially important issue for future research.

## Conclusion

Family function and supportive and non-supportive interactions within the family have implications for the patient with type 2 diabetes. The implications include adherence to the recommended lifestyle and the general well-being of the patient. Looking ahead, we propose that interventional studies that include the assessment of family function and professional support by family-based educational interventions will have the potential to improve the daily life and well-being of patients with type 2 diabetes and their family.
